# A Case of Placenta Prævia

**Published:** 1870-01

**Authors:** I. B. Washburn

**Affiliations:** Star City, Indiana


ARTICLE VI.—*A Case of Placenta Prævia.* Reported by I. B. WASHBURN, M.D., Star City, Indiana.

THE following case occurred in the practice of Dr. D. Rea, of Royal Center. Dr. Rea says that, August 17th, 1869, Mr. H. called at his office and asked him to prescribe for his wife, as she was flooding.

The doctor gave him opii pulv. gr. ss., plumbi acetas gr. iij, to be given every three hours until the hæmorrhage should cease. He heard nothing more from his patient until the 24th, when he was called to visit her because of a recurrence of the uterine hæmorrhage. He made an examination, and found the os slightly patulous and rather soft and flabby, but there were no symptoms of labor.

He repeated the opium and lead, which seem to control the hæmorrhage again.

The next morning he was called in great haste and found his patient flooding fearfully. He immediately dispatched a messenger for assistance, but before any arrived, the doctor, knowing that something must be done immediately in order to save his patient, passed his hand through the placenta and ruptured the membranes. The hæmorrhage ceased, the vertex prevented and pressed upon the placenta, compressing it so that a further discharge of blood seemed impossible while it remained in that condition. Upon my arrival the patient was anæmic, pulseless at the wrist, extremities very cold, and she was in a semi-conscious state. The ragged edge of the placenta could be felt around the head of the child, the os was well dilated, the occiput opposite the left acetabulum, but there were no contractions of the uterus. Nature seemed exhausted.

We gave her ammonia carb. and camphor freely, alternated with a decoction of ergot, in small doses. In a short time there were signs of reaction.

We then prepared to deliver by version, but upon introducing my hand I found the head engaged in the upper strait, and believing the fœtus dead because of the loss of blood, we increased the stimulants, both general and uterine, and soon saw our patient delivered of a large, still-born male child. There was no more hæmorrhage. We applied heat externally and gave her ammonia and alcoholic stimulants freely, internally, after which she reacted gradually and slept.

The next day she was attacked with puerperal mania, which lasted about three weeks, but she recovered finally.

Her recovery being slow, notwithstanding Dr. Rea gave her good attention, through the meddling of officious neighbors, they changed to one Osborn, who, if he ever graduated or even attended a course of lectures, did so in "Brush College" or one of the "Sums" of Cincinnati. He is a medical "bushwhacker." He labeled one of his bottles "femail pils," and says that morphia is made of "*minerals and combustibles.*"

